# Edentulism, Social Mobility, and Cognitive Aging: A Life Course Perspective

**DOI:** 10.1093/geroni/igaf122.1226

**Published:** 2025-12-31

**Authors:** Ruotong Mona Liu, Huabin Luo, Xiang Qi, Zhijing Xu, Bei Wu

**Affiliations:** New York University, New York, New York, United States; East Carolina University, Greenville, North Carolina, United States; New York University, New York, New York, United States; New York University, New York, New York, United States; NYU Shanghai, Shanghai, China (People’s Republic)

## Abstract

While edentulism and social mobility have each been linked to cognitive decline among older adults, few studies have examined their interactive effects or how these relationships differ across age groups. This study investigated the joint effects of edentulism and social mobility on cognitive function and age-specific patterns. We analyzed data from the Health and Retirement Study (2006–2020), comprising 23,158 adults aged 51+. Cognitive function was assessed using modified Telephone Interview for Cognitive Status. Social mobility was represented by intergenerational education mobility, and edentulism was determined through self-reporting. Linear mixed-effects models with lagged time-varying covariates were used to examine how these factors affected cognitive changes over time. At baseline, 13.2% of participants were edentulous. We classified participants into four social mobility groups: stable low socioeconomic position (SEP) (29.6%), stable medium/high SEP (34.3%), upward mobility (17.1%), or downward mobility (19.1%). Edentulism was associated with accelerated cognitive decline, whereas all non–low-SEP groups had higher baseline cognition. The stable medium-high SEP group also demonstrated slower cognitive decline and greater resilience against the negative effects of edentulism over time. The interaction effects between edentulism and social mobility were most pronounced in older adults aged 65-74, while neither tooth loss or social mobility significantly affected cognitive outcomes in those 75 and older. The results highlight that edentulism and social mobility play an important role in cognitive trajectories both independently and jointly, with effects varying across age groups. Targeting oral health, especially among adults aged 51–74, may help mitigate cognitive decline within diverse SEP.

